# Retrospective Long-Term Evaluation of Conventional Transarterial Chemoembolization for Hepatocellular Carcinoma over 20 Years

**DOI:** 10.3390/cancers16081498

**Published:** 2024-04-14

**Authors:** Thomas J. Vogl, Hamzah Adwan, Leonard Wolff, Maximilian Lahrsow, Tatjana Gruber-Rouh, Nour-Eldin Abdelrehim Nour-Eldin, Jörg Trojan, Wolf-Otto Bechstein, Nagy N. N. Naguib

**Affiliations:** 1 Clinic for Radiology and Nuclear Medicine, University Hospital Frankfurt, Goethe University, 60590 Frankfurt, Germany; 2 Department of Diagnostic and Interventional Radiology, Faculty of Medicine, Cairo University, Cairo 12613, Egypt; 3 Department of Gastroenterology, University Hospital Frankfurt, Goethe University, 60590 Frankfurt, Germany; 4 Department of General and Visceral Surgery, University Hospital Frankfurt, Goethe University, 60590 Frankfurt, Germany; 5 Radiology Department, AMEOS Hospital Halberstadt, 38820 Halberstadt, Germany; 6 Department of Diagnostic and Interventional Radiology, Faculty of Medicine, Alexandria University, Alexandria 21526, Egypt

**Keywords:** hepatocellular carcinoma, interventional radiology, interventional oncology, conventional transarterial chemoembolization

## Abstract

**Simple Summary:**

HCC is the most common malignant primary hepatic tumor, which can be treated by various therapies including surgery, ablation, or transarterial methods such as cTACE. Each of these treatments has its own indications depending on the stage of the disease. cTACE is mostly performed in patients with intermediate stage HCC. This large retrospective single-center study shows the long-term results including survival and prognostic factors of patients with HCC treated by TACE in different treatment settings over 20 years.

**Abstract:**

The aim of this study was to retrospectively evaluate the effects of conventional transarterial chemoembolization (cTACE) for the treatment of hepatocellular carcinoma over 20 years regarding overall survival (OS) and prognostic factors for OS. During the period from 1996 to 2016, 836 patients with HCC were treated with cTACE. Data evaluation was performed on the basis of pre- and postinterventional MRI and CT scans. Survival analysis was performed by Kaplan–Meier estimator; prognostic factors were determined by the use of Cox regression analysis. Overall, 4084 (mean 4.89 TACE sessions/patient) procedures were assessed. Median OS was 700 days (99% CI, 632.8–767.2). Depending on the indication, patients treated with a neoadjuvant intention showed the best OS (1229 days, 99% CI 983.8–1474.2) followed by curative intention (787 days, 99% CI 696.3–877.7), and then palliative intention (360 days, 99% CI 328.4–391.6). Portal vein thrombosis (HR 2.19, CI 1.63–2.96, and *p* < 0.01) and Child–Pugh class B or worse (HR 1.44, CI 1.11–1.86, and *p* < 0.001) were significantly associated with shorter OS. Patients with HCC benefit from TACE after careful patient selection. Portal vein thrombosis and Child–Pugh class B or worse are significantly unfavorable prognostic factors for patients’ survival.

## 1. Introduction

Hepatocellular carcinoma (HCC) remains the most common primary tumor of the liver [1], making up about 75–85% of primary liver cancers [2]. The five-year survival rate of patients with HCC is more than fifty percent in curative situations [3]. However, the incidences are increasing in the European countries, as well as Oceania, and the Americas [4]. HCC is the fifth most common cancer in men worldwide [5].

HCC has many risk factors, with the most frequent ones being an infection with a hepatotropic virus, mainly Hepatitis B and C Virus, chronic alcohol consumption, exposure to aflatoxin, or smoking [6,7].

The treatment of HCC mostly depends on the disease situation of the patient. Liver transplantation (LT) represents an optimal treatment for HCC [8]. Beneficial hereby is the fact that not only the HCC is treated, but also the underlying liver disease [8]. Other treatment methods include surgical resection, interventional treatments, for instance percutaneous ethanol injection (PEI), radiofrequeny ablation (RFA), microwave ablation (MWA), cryoablation, or transarterial chemoembolization (TACE) [9].

TACE is a first-line treatment for HCC patients with an intermediate stage of disease with well-defined tumors according to the latest version of the Barcelona Clinic Liver Cancer (BCLC) guideline [10]. TACE also several other indications and can be performed symptomatically or palliatively, [11] as well as in a combination treatment with thermal ablation [11,12] as shown by several studies [13,14,15,16,17,18,19,20] with very promising results. Patients with a BCLC stages of 0 or A can be treated in a curative intention by thermal ablation, resection, or LT [10].

TACE is based on the injection of chemotherapeutic and embolic agents in the tumor-supplying arteries [21]. Conventional TACE (cTACE) and drug-eluting bead (DEB)-TACE are the two main sorts of TACE [22,23]. cTACE is carried out by applying chemotherapeutical drugs which are emulsified in lipiodol, which is then followed by the application of embolizing material to halt the blood flow [24]. On the other hand, DEB-TACE is performed by the injection of DEBs in which the chemotherapeutic agent is fixated in the tumor arteries [12]. cTACE and DEB-TACE provide comparable outcomes [25].

While the subject of TACE for the management of HCC has been sufficiently addressed in the literature, to our knowledge, none of the available studies examined the very long-term outcome of TACE in a large patient cohort.

The purpose of this study is to retrospectively evaluate the outcomes of cTACE for the treatment of HCC over a period of twenty years mainly regarding overall survival (OS) and prognostic factors for OS.

## 2. Materials and Methods

### 2.1. Patients

This retrospective study was performed following the approval of the local ethics committee for all patients with HCC who underwent TACE between 1996 and 2016. Patients’ clinical information was extracted from the institute’s database, which contains all patients who received interventional procedures of the liver. All patients who underwent TACE due to HCC were included in this study, hence a total of 836 patients (653 men and 183 women; mean age: 74.18 years) were acquired. Hereby, exclusion criteria were age < 18 years or insufficient data. As radiological images were available going onwards from 2006, patients treated between 2006 and 2016 were additionally evaluated by size measurement of the index lesion in cm^3^. The included patients underwent multiple cycles each consisting of several separate sessions of TACE. We mainly focused on the OS as well as prognostic factors for OS. The evaluated parameters were patients’ Child–Pugh class, indication for TACE, size of spleen, presence of a portal vein thrombosis (PVT), and the applied chemotherapeutics.

Most of the included patients had Child–Pugh class A followed by Child–Pugh class B at rates of 59.21% and 22.49%, respectively. Eighty-six patients had complete PVT and fifty-seven had partial PVT. Otherwise, most of the included patients did not have PVT. The number of patients with splenomegaly was 365 at a rate of 43.66%.

A total of 4084 sessions of TACE were performed with a mean number of 4.89 sessions per patient. Four hundred sixty patients underwent ≤3 TACE sessions at a rate of 55% and two hundred and forty-three patients (29.1%) were treated by 4–6 sessions of TACE. The rest 133 patients (15.9%) underwent >6 TACE sessions.

Three hundred ninety-four patients were treated in a neoadjuvant setting and four hundred and thirty-one patients in either curative or palliative intentions. In 11 patients, the indication could not be elicited retrospectively. Of the patients with a neoadjuvant TACE, 112 patients received MWA after TACE, 78 patients were treated with laser induced thermotherapy (LITT), 49 patients underwent LT, 39 patients underwent surgical resection, and 34 were treated with RFA. Two patients underwent selective internal radiation therapy (SIRT). In a total of 80 cases, the subsequent therapy after TACE was unknown. Details of patients and treatments are summarized in Table 1.

### 2.2. Imaging Protocol

Cross-sectional imaging techniques including magnetic resonance imaging (MRI) and computed tomography (CT) examinations were used to perform the imaging studies. Non-enhanced and contrast-enhanced MR imaging was performed in all patients prior to the first interventional procedure. The pre-interventional MRI protocol included T1-weighted gradient-echo sequences (FLASH-2D) with transverse and sagittal slice orientation (TR/TE: 135/6 ms; FA 80°; FOV 350 mm; matrix 134 × 256; slice thickness 8 mm; interslice gap 0.8 mm). In addition, non-enhanced T2-weighted breath-hold turbo-spin-echo sequences (TR/TE: 3800/92 ms; FA 150°; FOV 350 mm; matrix 115 × 256; slice thickness 8 mm; interslice gap 0.8 mm) and contrast-enhanced dynamic VIBE sequences (TR/TE: 4.5/1.8 ms; FA 15°; FOV 350 mm; matrix 128 × 256; slice thickness 8 mm; interslice gap 0.8 mm) were performed. The patients underwent CT scans mainly after the TACE sessions to evaluate the distribution of lipiodol within the liver and detect possible spreading into other organs. Further follow-up examinations were conducted using MRI directly before the next TACE session.

### 2.3. TACE Technique

All patients were informed of the risks, side effects, and alternative therapeutic options at least 24 h before therapy and informed consent was obtained. After sterile covering of the inguinal region and the injection of a local anesthetic, a catheter was introduced through the femoral artery using the Seldinger technique [26]. A 5F-pigtail catheter was first used to perform angiography to visualize the vessels supplying the tumor, and then a switch was made to a 5F sidewinder catheter via the guide wire. This catheter was then used to selectively catheterize and visualize the superior mesenteric artery and the coeliac trunk. Following that, a catheter was proceeded into the supplying arteries of the tumor. After reaching the target position, the application of the chemotherapeutic agent was initiated, as well as the subsequent application of the embolic materials. Once embolization was complete, sufficient occlusion of the vessels was checked by means of another angiography.

We used various chemotherapeutic drugs while performing TACE. However, mitomycin C, gemcitabine, and cisplatin were mainly used. These anticancer drugs were applied alone or in combination with each other. The occlusion of the targeted vessels was accomplished with a maximum of 10 mL of lipiodol, followed by an injection of 60–180 mg of microspheres. Application of a compression bandage or a percutaneous closure device on the site of the puncture concluded the procedure.

The decision to perform further TACE sessions was made depending on the tumor response. Usually, the number of required procedures was a minimum of three interventions. In case of a tumor response or stable disease situation, further TACE sessions were performed. However, if a disease progression occurred after two successive TACE sessions, no further sessions were usually performed.

### 2.4. Statistical Analysis

Statistical analysis was performed using “BiAS.” version 11.08. *p*-values < 0.05 were considered statistically significant. OS was determined by the Kaplan–Meier estimator. Prognostic factors concerning the risk of death were determined by the Cox regression analysis. All patients were divided into four groups, each covering an interval of five years.

## 3. Results

### 3.1. Overall Survival

**Figure 1:**
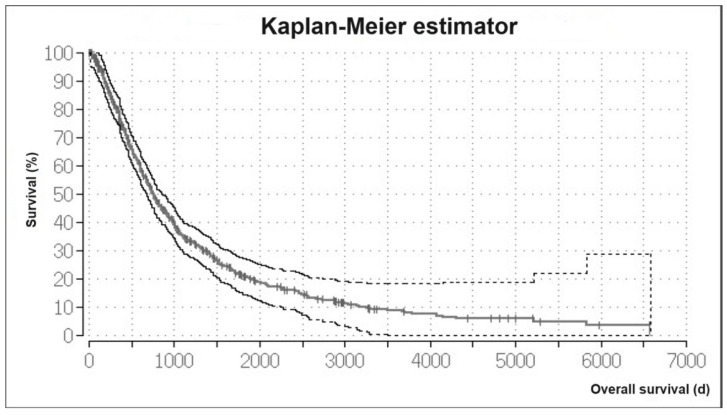
Kaplan–Meier estimator. Overall survival of HCC patients with interventional treatment.

The mean OS time was 1223.76 days and the median OS time was 700 days (99% CI, 632.8–767.2) for all treated patients. The Kaplan–Meier analysis of the OS showed a 1-year survival rate of 80%. The 5-year survival rate was 21%. The Kaplan–Meier curve for all patients is shown in Figure 1.

Survival analysis regarding different indications showed that the median OS time in patients with a neoadjuvant indication was 1229 days (99% CI, 983.8–1474.2), and the mean OS time was 1700 days. Patients with a curative indication had a median OS time of 787 days (99% CI, 696.3–877.7) and a mean OS time of 1048 days. Patients in a palliative setting had a median OS time of 360 days (99% CI, 328.4–391.6) and a mean OS time of 429.9 days.

**Figure 2:**
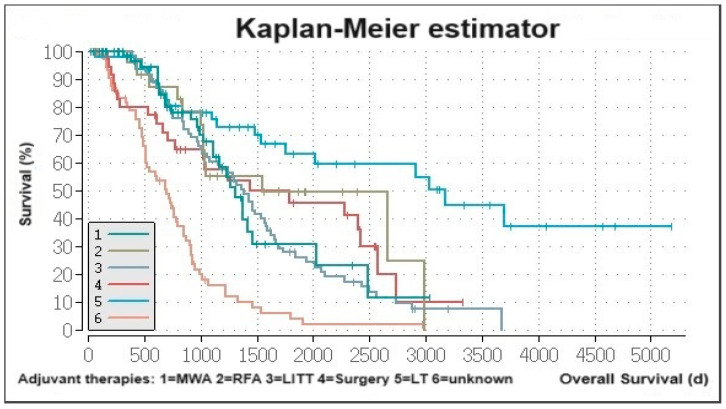
Survival in patients with neoadjuvant indication sorted by their subsequent therapy after TACE.

Survival analysis for neoadjuvant treated patients sorted by their definitive treatment showed that patients who received LT had a median OS time of 3175 days (95% CI 1682.5–4667.5). Those treated with MWA, RFA, LITT, and surgery had similar OS times (MWA: 1308 days, 95% CI 1082.1–1533.9; RFA: 1545 days, 95% CI 416.7–2673.3; LITT: 1385 days, 95% CI 1174.3–1595.7; surgery: 1437 days, 95% CI 89.7–2784.3). Patients with unknown treatment post-TACE had a median OS time of 693 days (95% CI 540.1–845.9) (Figure 2).

### 3.2. Determination of Predictors for Survival

Cox regression analysis was performed to investigate whether there are predictors for death due to HCC. Examined parameters were sex, Child–Pugh class, splenomegaly, presence of PVT, and the number of treatment sessions. Those parameters were dichotomized as follows: men and women, average sized (<12 cm) versus enlarged spleen (≥12 cm), no PVT versus partial or complete PVT, Child–Pugh class A versus Child–Pugh classes B and C, and the number of treatment sessions in patients with 1–6 sessions versus ≥7 sessions. The relative hazard ratios (HR) regarding the parameters sex, splenomegaly, and number of treatment sessions were not statistically significant. The relative HR of existing PVT was 2.19 (CI 1.63–2.96 and *p* < 0.01), and the relative HR of Child–Pugh class B or worse was 1.44 (CI 1.11–1.86 and *p* < 0.001).

### 3.3. Evaluation of Tumor Volume Reduction

**Figure 3:**
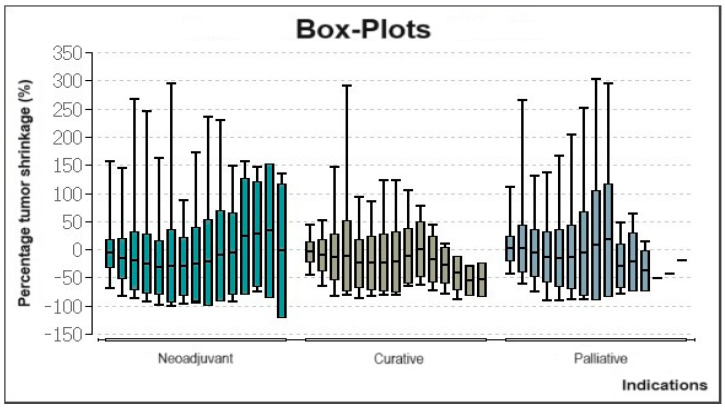
Box-plots showing percentage changes in tumor volume for all patients with HCC treated during the period of 2006 until 2016.

The percentage change in tumor volume was evaluated in a total of 358 patients with HCC treated between 2006 and 2016. Box-plots of this data show that treatment in neoadjuvant scenarios led to tumor shrinkage especially up until the 6th or 7th treatment session (Figure 3). Patients treated in a curative intention showed a similar course. All patients, regardless of indication, experienced an increase in tumor volume after at least the 9th consecutive interventional treatment.

### 3.4. Chemotherapeutics

The data regarding the used chemotherapeutics was available for 96% (3921/4084) of the performed TACE sessions. In the period between 1996 and 2001, the most used chemotherapeutic agent was mitomycin C alone at a rate of 81.6% followed by the combination of mitomycin C and gemcitabine at a rate of 10.8%.

In the period between 2002 and 2006, the combination of mitomycin C and gemcitabine was most frequently applied at a rate of 45.1% followed by mitomycin C alone at a rate of 39.8%.

In the period between 2007 and 2011, the most used chemotherapeutic agent was mitomycin C alone at a rate of 31.5% followed by the combination of mitomycin C, gemcitabine, and cisplatin at a rate of 27.3%.

Lastly, in the period between 2012 and 2016, the most commonly used chemotherapeutic agent was the combination of mitomycin C, gemcitabine, and cisplatin at a rate of 41.8% followed by mitomycin C alone at a rate of 27.6%. It can be concluded that over time, the application of mitomycin C alone was regressive, whereas the use of mitomycin C combined with cisplatin and gemcitabine increased. The comparison of the usage of the chemotherapeutic agents within the aforementioned periods is shown in Table 2.

## 4. Discussion

TACE is the most common treatment for HCC among all stages, especially in Europe and North America [27]. The most common second treatment for HCC after surgical resection in Europe is ablation using RFA/PEI followed by TACE [27]. TACE is the treatment of choice for patients with intermediate disease stage (BCLC B). These patients have a survival time of 16 months without treatment and 20 months when treated by TACE [28].

In this study, we investigated the long-term outcome of cTACE for HCC over 20 years in a large cohort and evaluated the prognostic factors for survival time. We could report a mean OS time of 1223.76 days (40 months) and a median OS time of 700 days (23 months) for all 836 patients. The associated 1- and 5-year survival rates in this study were 80% and 21%, respectively.

There are many studies that investigated TACE as a treatment for HCC in different settings. For example, Kong et al. [29] and Akarapatima et al. [30] found that TACE significantly improves the survival time of HCC patients compared to the best supportive care. In our study the best survival times were reported in patients treated neoadjuvantly with TACE, followed by other treatments including ablation and surgery. A systemic review and meta-analysis by Huo et al. compared the combination of radiotherapy and TACE with TACE alone for HCC [31]. They found that the combination therapy of both modalities was superior to TACE alone. Su et al. investigated in their retrospective study the long-term survival of HCC patients with macroscopic vascular invasion, who were treated by the combination therapy of TACE and radiotherapy compared to radiotherapy alone [32]. They found that the combination therapy was significantly superior regarding OS and progression-free survival in comparison to radiotherapy alone. Yang et al. showed in their systematic review and meta-analysis that OS and PFS are significantly improved when TACE and ablation are combined [33], supporting our findings that the best OS is accomplished when TACE is combined with adjuvant ablation or surgery. Parikh et al. [34] showed that almost half of all included patients outside the Milan criteria could be successfully downstaged. Further, they could show that multimodal locoregional therapy concept had significantly higher downstaging success rates compared to transarterial radioembolization/TACE. These studies provide further evidence of the benefits of multimodal therapy concepts. On the other side, patients undergoing interventional treatment with a palliative indication had the worst results as shown in this study. The studies by Mao et al. [35] and Wu et al. [36] who could both show that high tumor burden and poor function of liver have an effect on OS and prognosis of patients with HCC. Nevertheless, TACE should not be withheld from other indications, as the multimodal use of TACE, for instance in a palliative setting, showed promising results as well.

The conducted Cox regression analyses underlined that the stated pre-interventional patient selection could be of great value as we could prove that patients with HCC and PVT or a Child–Pugh class B or worse had a significantly higher risk of death, leading to the conclusion that pre-interventional patient selection should especially be focused on the patient’s Child–Pugh class and presence of PVT to improve therapy results. Similarly, Chang et al. [37] included in their study 108 patients with small HCCs who were treated by interventional treatments. They showed that the survival rates in patients with Child–Pugh class B were significantly worse compared to patients with Child–Pugh class A (HR = 2.68 and 95% CI = 1.52–4.73). Furthermore, Liu et al. investigated TACE for advanced HCC patients with PVT [38]. They included a total of 188 patients and found that amongst others, Child–Pugh class (HR = 2.981, 95% CI: 1.919–4.631, and *p* < 0.001) and PVT (HR = 2.806, 95% CI: 2.024–3.890, and *p* < 0.001) were independent predictors of survival.

This study has several limitations that should be considered. Firstly, the retrospective nature of the study. Secondly, the heterogeneity of the patients included in the current study and the non-standardization of the type and regime of the used chemotherapeutic agents. Thirdly, the study did not evaluate occurring complications as well as progression-free survival. Lastly, imaging studies of patients treated between 1996 and 2005 were not available and therefore, the evaluation was based on the written radiological reports.

## 5. Conclusions

In conclusion, this study demonstrates the possibilities and limitations of TACE as patients profit from TACE especially in the medium term, for instance, as part of neoadjuvant multimodal strategies. Though, the effectiveness is reduced in the long term. Additionally, our results underline the importance of pre-interventional patient selection as patients with PVT or Child–Pugh class B or worse tend to have poorer outcomes and survival.

## Figures and Tables

**Table 1:** Summarization of relevant characteristics of patients and treatments.

Characteristic	
Total number of patients	836
Mean age in years	74.18
Sex	
Male (%)	653 (78.1%)
Female (%)	183 (21.9%)
Child–Pugh class (%)	
A	495 (59.21%)
B	188 (22.49%)
C	122 (14.6%)
Unknown	31 (3.7%)
Portal vein thrombosis	
Complete	86 (10.3%)
Partial	57 (6.8%)
None	629 (75.2%)
Unknown	64 (7.7%)
Mean spleen size	12.8 cm
Splenomegaly (size ≥ 12.0 cm)	365 (43.66%)
Total number of cTACE	4084
Mean number of TACE sessions/ patient	4.89
Patients with ≤3 TACE performed	460 (55%)
Patients with 4–6 TACE performed	243 (29.1%)
Patients with >6 TACE performed	133 (15.9%)
Indications	
Neoadjuvant	394 (47.1%)
Curative or Palliative	431 (51.6%)
Unknown	11 (1.3%)
Interventions/treatments after neoadjuvant TACE	
Microwave ablation	112 (28.43%)
Radiofrequency ablation	34 (8.63%)
Laser-induced thermotherapy	78 (19.8%)
Selective internal radiation therapy (SIRT)	2 (0.5%)
Surgical resection	39 (9.9%)
Liver transplantation	49 (12.44%)
Unknown	80 (20.3%)

**Table 2:** Listing of the most frequently used chemotherapeutic agents in chronological order.

Chemotherapeutic Regime	1996–2001	2002–2006	2007–2011	2012–2016
Mitomycin C Gemcitabine Cisplatin	6 (1%)	44 (3.4%)	342 (27.3%)	308 (41.8%)
Mitomycin C Cisplatin	3 (0.5%)	19 (1.4%)	245 (19.6%)	183 (24.9%)
Mitomycin C	506 (81.6%)	522 (39.8%)	395 (31.5%)	203 (27.6%)
Mitomycin C Gemcitabine	67 (10.8%)	592 (45.1%)	156 (12.4%)	7 (1%)
Other combinations/ chemotherapeutics	38 (6.1%)	135 (10.3%)	115 (9.2)	35 (4.7%)
∑ (%)	620 (100%)	1312 (100%)	1253 (100%)	736 (100%)

## Data Availability

The data may be requested from the corresponding author. All requests must be reasonable.
